# Effects of Antibacterial Peptide F1 on Bacterial Liposome Membrane Integrity

**DOI:** 10.3389/fnut.2021.768890

**Published:** 2021-11-12

**Authors:** Qun Wang, Bo Peng, Mingyue Song, Abdullah, Jun Li, Jianyin Miao, Konglong Feng, Feilong Chen, Xiaoxiang Zhai, Yong Cao

**Affiliations:** ^1^College of Food Science, South China Agricultural University, Guangzhou, China; ^2^Guangdong Haitian Innovation Technology Co., Ltd., Foshan, China; ^3^Evonik Rexim Nanning Co., Ltd., Nanning, China; ^4^Shanghai Seventh People's Hospital, Shanghai, China

**Keywords:** quartz crystal microbalance with dissipation (QCMD), antimicrobial peptide F1 (AMP), phospholipid membrane, antibacterial mechanism of action, bacteria

## Abstract

Previous studies from our lab have shown that the antimicrobial peptide F1 obtained from the milk fermentation by *Lactobacillus paracasei* FX-6 derived from Tibetan kefir was different from common antimicrobial peptides; specifically, F1 simultaneously inhibited the growth of Gram-negative and Gram-positive bacteria. Here, we present follow-on work demonstrating that after the antimicrobial peptide F1 acts on either *Escherichia coli* ATCC 25922 (*E. coli*) or *Staphylococcus aureus* ATCC 63589 (*S. aureus*), their respective bacterial membranes were severely deformed. This deformation allowed leakage of potassium and magnesium ions from the bacterial membrane. The interaction between the antimicrobial peptide F1 and the bacterial membrane was further explored by artificially simulating the bacterial phospholipid membranes and then extracting them. The study results indicated that after the antimicrobial peptide F1 interacted with the bacterial membranes caused significant calcein leakage that had been simulated by different liposomes. Furthermore, transmission electron microscopy observations revealed that the phospholipid membrane structure was destroyed and the liposomes presented aggregation and precipitation. Quartz Crystal Microbalance with Dissipation (QCM-D) results showed that the antimicrobial peptide F1 significantly reduced the quality of liposome membrane and increased their viscoelasticity. Based on the study's findings, the phospholipid membrane particle size was significantly increased, indicating that the antimicrobial peptide F1 had a direct effect on the phospholipid membrane. Conclusively, the antimicrobial peptide F1 destroyed the membrane structure of both Gram-negative and Gram-positive bacteria by destroying the shared components of their respective phospholipid membranes which resulted in leakage of cell contents and subsequently cell death.

## Introduction

The phospholipid bilayer is the foundation of all bacterial cell membranes and antimicrobial peptides usually target this membrane to kill bacteria (1, 2). Therefore, the mechanism of action between an antimicrobial peptide and the phospholipid membrane is the core of the peptide's ability to penetrate into the membrane (3). Single or multiple phospholipids are used to make liposomes, which are similar to the phospholipid membranes of microorganism to simulate the biofilm and study the mechanisms associated with the antimicrobial peptides (4). Among these models, liposome, solid supported, and micelle are all classical methods to study the interaction between various antimicrobial substances and the phospholipid membrane.

In water, phospholipids or other similar compounds actively form a closed structure called a liposome. The chemical composition and thickness of the formed liposomes are very similar to those of the natural membranes. Liposomes also have fluidity and asymmetry, so they are widely used as a membrane model (5). Liposomes made of either natural or synthetic phospholipids can simulate different kinds of cell membranes by changing their phospholipid composition. For instance, there is a higher content of phosphatidyl ethanolamine (PE) in the membrane of Gram-negative bacteria such as *E. coli* (6). In addition, bacterial cell membranes also contain phosphotidyl glycerol (PG), cardiolipin (CL), and other negatively charged phospholipid molecules. However, the major phospholipid molecules found in the mammalian somatic cell membrane are phosphatidyl choline (PC) and cholesterol (CHOL) (7). A mixture of PG and PE phospholipids has been used to simulate the cell membrane of *E. coli*; some people have also used PC, PG, and CHOL to simulate the surface of Gram-positive bacterial cell membranes (8). Dipalmitoylphosphatidylcholine (DPPC), dipalmitoylphosphatidylglycerol (DPPG) and 1,2-Dipalmitoryl-sn-Glycero-3-Phosphoethanolamine (DPPE) are important components of PC, PG, and PE, respectively, and are often used to simulate phospholipid membranes. To explore the effects of antimicrobial peptides on membrane permeability, liposomes have been embedded with fluorescent probes such as calcein to study the leakage of their contents (9). When different concentrations of antimicrobial peptides applied on calcein-containing liposomes which resulted in changing phospholipid membrane permeability due to the actions of the antibacterial peptide. This permeability change was evident from the release rate of calcein (10). Solid supported membrane is a membrane model of the phospholipid membrane that is spread on a plane and simulates the basic, biological structure of the phospholipid bilayer (1). Quartz Crystal Microbalance with Dissipation (QCM-D) monitors the frequency (F) and energy dissipation (D) of the quartz crystal surface in real time, thereby monitoring the dynamics of the membrane solid support membrane surface, and measuring the quality and viscoelastic changes in the phospholipid membrane (11, 12). Moreover, due to their different sound penetration depths, higher octave frequencies monitor the information on the surface of the sensor, while lower octave frequencies are closer to the water surface. Therefore, QCM-D is used to monitor the quality and viscoelastic changes at different depths of the phospholipid membrane on the surface of the quartz crystal in real time. This information allows one to preliminarily infer the types of interactions between the antimicrobial peptide molecules and the phospholipid membrane (13, 14).

In our previous research, which reported an antimicrobial peptide F1 (Thr-DAP-Asn-Thr-PEA-Gln-Ala-Arg-Ser-Lys-Gln-Asp-PEA-CySH-Val-Asn-PEA-Tau) (15), a novel antimicrobial peptide obtained from Tibetan kefir. Briefly, this work showed that the antimicrobial peptide F1 had strong antimicrobial activity against several bacterial and fungal strains (16, 17). Based on this initial work, present study aimed to determine the destructive effects of antimicrobial peptide F1 on the bacterial membrane structure particularly. In this study, transmission electron microscope (TEM) was used to investigate the changes in the bacterial membrane microstructure after the antimicrobial peptide F1 acted on both *E. coli* and *S. aureus* and caused leakage of potassium and magnesium ions. Secondly, different liposomes (DPPG, DPPE, DPPC, and CHOL) to simulate various bacterial phospholipid membrane compositions followed by calcein permeability, TEM, QCM-D, and other methods to study the interaction between the antimicrobial peptide F1 and bacterial phospholipid membrane.

## Materials and Methods

### Microorganisms and Materials

Probiotic *L*. *paracasei* subsp. tolerans FX-6 was used in the present study, previously isolated from Tibetan kefir (traditional fermented milk from Tibet, China), and stored at −80°C in the College of Food Science, South China Agricultural University, Guangzhou, China. Both *Escherichia coli* ATCC 25922 and *Staphylococcus aureus* ATCC 63589 were stored at −80°C in the microbial culture laboratory. DPPG, DPPE, DPPC, and CHOL were acquired from Corden Pharma (Liestal, Switzerland). Cholesterol and calcein were purchased from Sangon Biothch (Shanghai, China). All the solvents and chemicals used in this study were of analytical grade.

### Transmission Electron Microscopy Observations of the Damaged Bacterial Membrane by F1

The tested bacterial strains used in this study *E. coli* and *S. aureus* were liquid cultured to the late logarithmic growth stage. After reaching this stage, the antimicrobial peptide F1 was added at a final concentration of 5 × MIC, after which it was cultured for 2 h at 37°C and sampled at 0.5, 1, and 2 h. Afterwards, centrifugation (2,000 rpm/min) for 10 min was used to collect the cells, after which the following steps were taken; (1) three rinses (10 min/rinse) with 5 mmol/L PBS buffer; (2) fixation with osmium acid for 1 h; (3) three rinses (10 min/rinse) with 5 mmol/L PBS buffer; (4) dehydration using a series of ethanol concentrations (30, 50, 70, 80, 90, and 100%). Among these, dehydration steps using 30, 50, 70, and 80% ethanol lasted for 10 min; cells were dehydrated with 90 and 100% ethanol twice per concentration (10 and 15 min/step, respectively); (5) dehydration using 100% acetone (twice, 15 min/step) (6) soaking in acetone: EPON812 resin in a 3:1 ratio for 2 h; (7) soaking in acetone:EPON812 resin in a 1:1 ratio overnight; (8) soaking in acetone:EPON812 resin in a 1:3 ratio for 6 h; (9) soaking in pure EPON812 resin twice for 12 h each time; (10) polymerize at 45°C for 24 h; (11) polymerize at 60°C for 48 h; (12) ultrathin sectioning followed by staining with 3% uranyl acetate and imaging using TEM (18).

### Detection of Bacterial Cell Ion Leakage

One milliliter of bacteria (*E. coli* and *S. aureus*) was removed and cultured to the late logarithmic growth stage. After reaching this stage, it was centrifuged, washed twice with sterile ultrapure water, and then 950 μL of sterile ultrapure water and 50 μL of antimicrobial peptide F1 were added. The final concentration was 1 × MIC. Ultrapure water was used as a negative control, and the antimicrobial peptide Triton X-100 was used as a positive control. Samples were incubated for different times (30, 60, 90, 120, 150 min) in a water bath (37°C) and then centrifuged (4,000 rpm/min, 10 min). After centrifugation, 0.1 mL of the supernatant was removed and 9.9 mL of ultrapure water was added to dilute it. An atomic absorption spectrometer was used to detect the concentration of potassium and magnesium ions.

### Extraction and Preparation of Lipids From *E. coli* and *S. aureus*

Lipid extraction was based on the method used by Dennison et al. (19) with minor modifications. Briefly, the bacterial solution that had been cultured to the logarithmic growth phase was placed in a 50 mL centrifuge tube and centrifuged at 4,000 rpm/min for 15 min at 4°C. The supernatant was re-suspended by adding PBS buffer, and then centrifuged at 4,000 rpm/min for 10 min, after which the supernatant was discarded. A small amount of buffer was added to the pellet and cells were transferred to a 300 mL beaker. Then 60 mL of buffer and 180 mL of chloroform methanol (1:2, v/v) solution were added and the resulting solution was stirred for 90 min. The liquid was then transferred from the beaker to a 500 mL separatory funnel, after which it was allowed to stand for separation of the different layers. The lowest liquid layer was collected and placed in a 50 mL round bottom flask and the solvent was evaporated at 40°C. The remaining organic solvent was blown off using nitrogen, after which 5 mL of PBS buffer was added to the extracted phospholipids. The sample was then sonicated for 30 min in a water bath to disperse the adherent film in the buffer. Using a 300 W intermittent ultrasonication for 5 min, the liquid nitrogen was repeatedly frozen and thawed for 8 cycles, after which it was repeatedly extruded for 10 times with a 100 nm film of liposome extruder to evenly distribute the individual liposomes. From this liposome dispersion, 3 mL was taken and subjected to a particle size analyzer, three cycles at a time. After determining the initial particle size, the liposomes were separated into the following two groups: (1) control group (37°C for 10 min) and (2) experimental group (1 mg/mL F1 and placed at 37°C for 10 min). A particle size analyzer was then used to detect the resulting particle sizes; each group had three samples run in parallel and the experiment was repeated three times independently.

### Effect of Antimicrobial Peptide F1 on Artificial Liposomes Embedded With Calcein

Briefly, 10 mg of phospholipid was weighed and the phospholipid composition of the four groups was as follows: (1) 100% DPPG, (2) 30% DPPG and 70% DPPE, (3) 100% DPPE, (4) 90% DPPC and 10% cholesterol. To each group, 5 mL of chloroform was added to fully dissolve the included phospholipids and a 50 mL round-bottom flask was used to evaporate to dryness at 40°C. The remaining organic solvent was blown off using nitrogen to form a film on the bottom of the flask. Five milliliter of calcein solution (60 mM) was added to the above-mentioned flask, after which the solution was fully hydrated at 60°C and probed using 300 W intermittent ultrasound for 5 min. The solution was then filtered 8–10 times using a 100 nm filter to obtain a mixture of liposomes with a uniform particle size. From this mixture, 1 mL was removed and passed through a 1.5 × 60 cm dextran gel G-50 column to remove any free calcein from the liposomes. The mobile phase uses PBS buffer, and each 4 mL sample is collected in a test tube separately. Then, a multi-functional microplate reader was used to separately detect the fluorescence value of the liquid in each tube. The excitation and emission wavelengths were 485 and 530 nm, respectively.

To this, 10% Triton X-100 was added and mixed thoroughly prior to re-measuring. The sample with the largest fluorescence change before and after adding Triton X-100 was selected for the following experiment. The calcein-embedded liposome fluid was added to a 96-wells plate (200 μL per well) and its fluorescence value was detected (excitation and emission wavelengths were 485 and 530 nm, respectively). Afterwards, 10 μL of different concentrations of F1 (0, 20, 40, 60, and 120 μM) solution was added to each group. Fluorescence was measured after every 2 min. Finally, 10% Triton X-100 was added and thoroughly mixed to determine the final fluorescence value to indicate complete leakage. The following formula was then used to calculate the different fluorescence leakage values: Leakage % = (F_t_ – F_0_)/(F – F_0_) × 100%, where F is the fully leaked fluorescence value, F_0_ is the initial fluorescence value, and Ft is the measured fluorescence value (20, 21).

### Using QCM-D to Study the Effect of F1 on Spreading Phospholipid Membrane

Liposome (70% is DPPG and 30% is DPPE) sample was weighed (1 mg/mL) and prepared into a liposome solution according to the previously described method in section Effect of Antimicrobial Peptide F1 on Artificial Liposomes Embedded With Calcein. The surface of the quartz wafer was cleaned with alcohol, then dried with nitrogen and loaded into the instrument. The samples were then sequentially introduced into the crystal chamber. The operating procedure was as follows: (1) 0–20 min (buffer used to make a stable baseline); (2) 20–30 min (rinse with 1 mg/mL liposome solution at a flow rate of 100 μL/min); (3) 30–40 min (pump stopped and solution was allowed to stand; the liposomes were fully ruptured and spread; (4) any liposomes that were not adsorbed onto the wafer were washed away with buffer at a flow rate of 100 μL/min for 40–50 min; (5) antibacterial peptide F1 (50 μL/min) was flowed over the wafer for a 50–60 min rinse; (6) 60–90 min (no solution flow and F1 was allowed to fully work on the membrane); (7) ~90–100 or 90–130 min (unadsorbed and detached components were washed away with buffer at a flow rate of 100 μL/min) (22).

### TEM Observations of Liposome Morphology Before and After F1 Treatment

High-purity antibacterial peptide F1 (1 mg) was added and mixed well into the liposome solution prepared according to the previously described method in section Detection of Bacterial Cell Ion Leakage. Samples were removed from this liposome solution at 1 and 10 min before F1 addition. TEM sample preparation was as follows: The treated liposome suspension was added dropwise to a 200-mesh carbon support membrane and dried overnight at 40°C. Then, 2% phosphotungstic acid was added dropwise to negatively stain the sample. After 1 min, the dye was sucked away and dried. Then, the prepared carbon support film was observed by TEM (23).

### Statistical Analysis

All assays were conducted in triplicate and the average values were taken for data analysis. The data was expressed as mean values ± standard deviation (SD). The statistical significance was measured using one-way analysis of variance (ANOVA) and a multiple comparison by Tukey's test. Moreover, statistically significant difference was determined at *P* < 0.05. Standard curves and other figures were created using GraphPad Prism 5.0 (GraphPad Software Inc., San Diego, CA, USA) and Origin 8.5 [OriginLab (Microcal), CA, USA].

## Results and Discussion

### Microscopic Morphology Effect of Antimicrobial Peptide F1 on *E. coli* and *S. aureus* Cells

**Figure 1:**
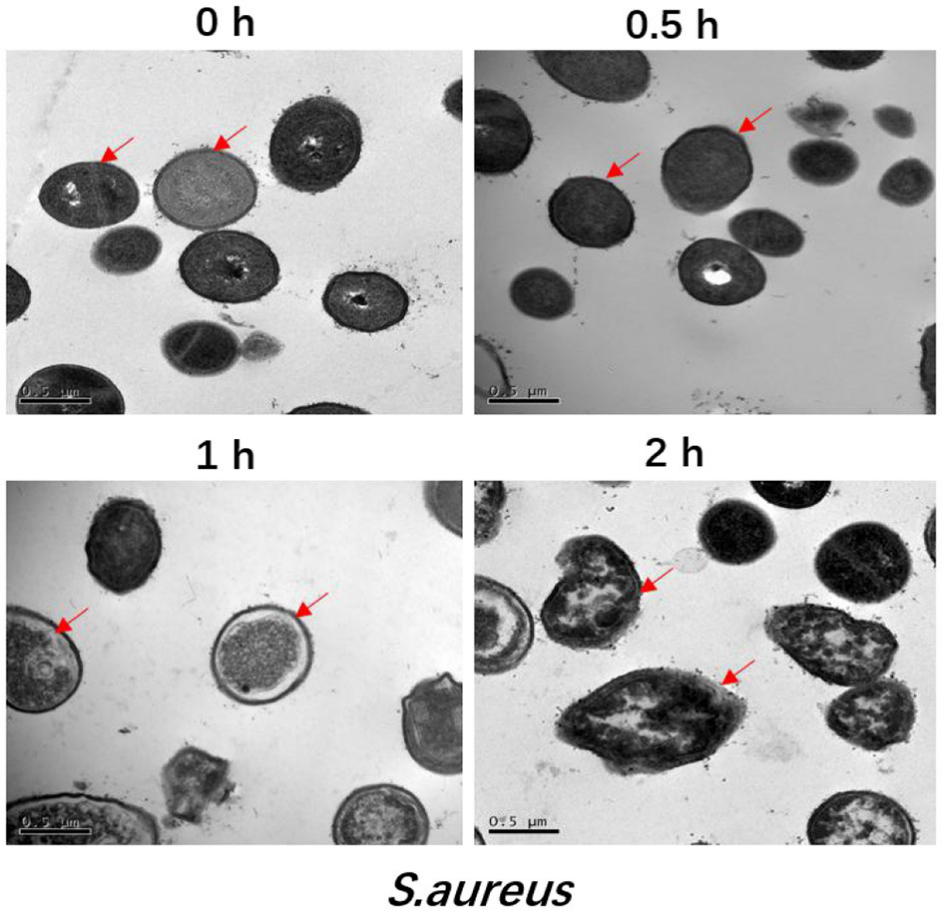
Transmission electron microscopic analysis of *S. aureus* treated by 5 × MIC of antimicrobial peptide F1 for 0, 0.5, 1, and 2 h, respectively. All images are representative of three independent experiments. The arrow is pointing a breakage in the membrane.

The ultrastructural changes of *S. aureus* over time after addition of the antimicrobial peptide F1 are shown in Figure 1. Normal *S. aureus* at time 0 h and without the effects of antimicrobial peptide F1 on its cell wall and membrane are clearly visible. The cell morphology is the typical morphology of *S. aureus* with a uniform cytoplasm, clear and complete cell wall and cell membrane, and smooth surface. After 0.5 h of incubation with antimicrobial peptide F1, the surface of the *S. aureus* cell membrane became rougher, with blurred cell wall and cell membrane and an uneven cytoplasm. After 1 h incubation with the antimicrobial peptide F1, the *S. aureus* cell membrane was almost mixed with the entire cell and the surface contour became blurred. The cell morphology was notably deformed and normal cells were not present, indicating that the intracellular lysate had leaked due to rupturing of cells and a complete dried out phenomenon occurred. After 2 h incubation with the antimicrobial peptide F1, the morphology of *S. aureus* cells was severely deformed and the cells were severely lysed, making it impossible to identify the cellular morphology.

Antimicrobial peptide F1 was also applied and investigated against *E. coli*, and the results had been published in a previous paper (15). Similar to the results observed in present study against *S. aureus*, the *E. coli* membrane was also gradually damaged after addition of the antimicrobial peptide F1. These result indicate that the antimicrobial peptide F1 has the potential to simultaneously damage the membranes of Gram-positive and Gram-negative bacteria (24). The main components of Gram-positive bacterial membrane are peptidoglycan, periplasmic space, and plasma membrane. The cell membrane of Gram-negative bacteria is composed of an outer membrane (lipopolysaccharide and protein), periplasmic space, and plasma membrane (25). Gram-positive bacteria have a thick peptidoglycan layer and no outer lipid membrane; comparatively, Gram-negative bacteria have a thin peptidoglycan layer along with an outer lipid membrane (26). Given this, the antimicrobial peptide F1 is likely to destroy the membrane structure by destroying the plasma membrane components shared by both Gram-positive and Gram-negative bacteria.

### Bacterial Ion Leakage After Antimicrobial Peptide F1 Acts on *E. coli* and *S. aureus*

**Figure 2:**
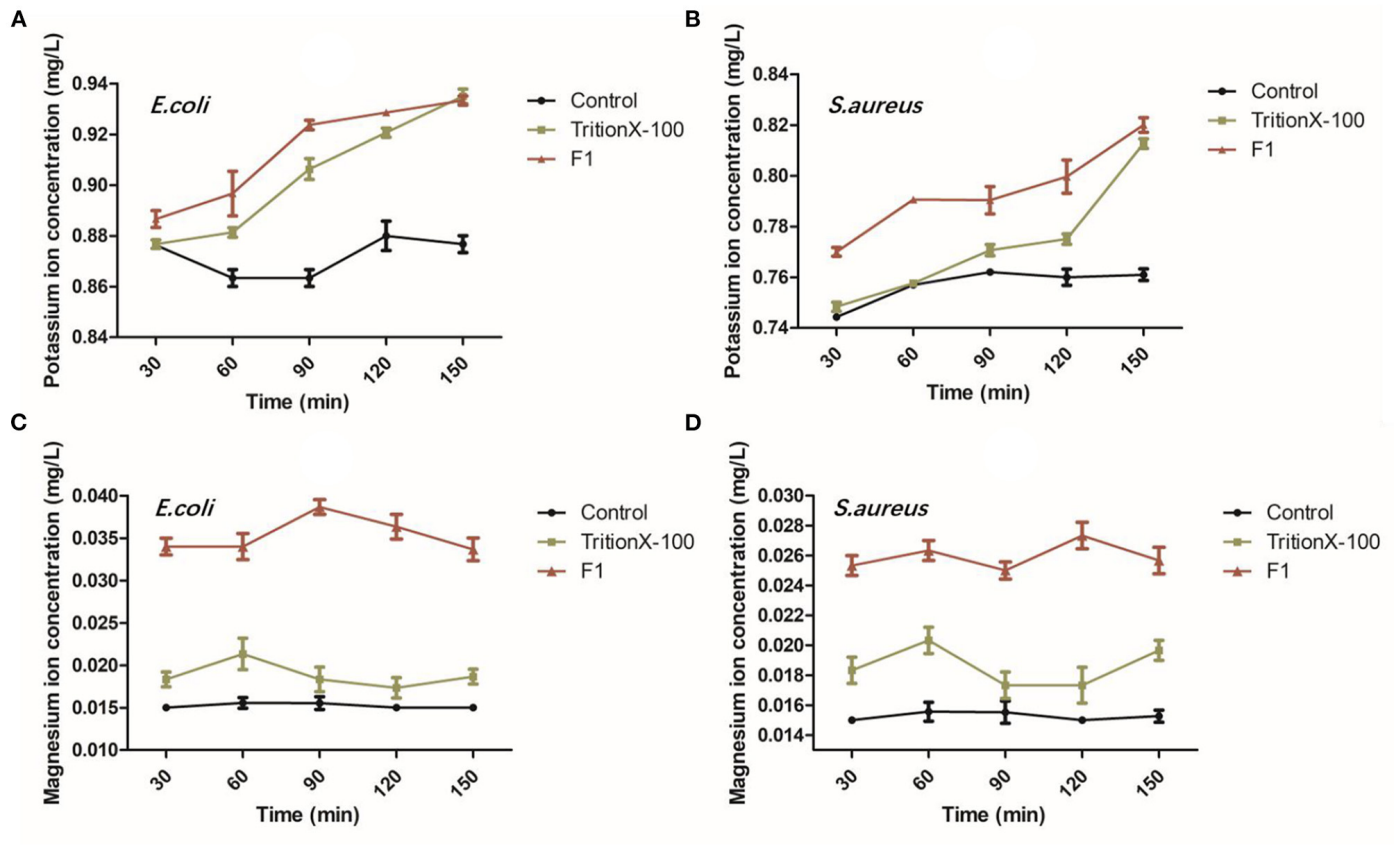
Analysis of bacterial ion leakage after antimicrobial peptide F1 applied on *E. coli* and *S. aureus*. **(A,C)** As well as **(B,D)** show the leakage of potassium and magnesium ions after the antimicrobial peptide F1 acted on *E. coli and S. aureus* for 30, 60, 90, 120, and 150 min, respectively.

The potassium ion leakage from *E. coli* showed an increasing trend from 0 to 90 min and at the initial concentration of ions in F1 group was found higher than other groups. These results showed that F1 can contact and destroy the bacterial membrane in a short time (<30 min). In all groups, the concentration of potassium ion reached the maximum in 30 min and remained relatively stable, indicating that potassium ion is very sensitive to bacterial membrane damage. The potassium ion concentration for *S. aureus* continued to rise slowly during the first 120 min, while the potassium ion leakage rate suddenly increased after 120 min. Our results also indicated that the antibacterial peptide F1 had a stronger effect on both bacteria in the first 150 min than the membrane breaker Triton X-100. After 150 min, the ion leakage concentrations of the antimicrobial peptide F1 and Triton X-100 treatment groups were similar (Figures 2A–D). Compared with the negative control, F1 caused a notably larger amount of magnesium ion leakage in the somatic cells of *E. coli* and *S. aureus*. Moreover, the effect was better than that of the positive control of Triton X-100.

Some studies showed that most of the antimicrobial peptides from lactic acid bacteria form holes in the bacterial cell membrane, causing intracellular ions to leak out and ultimately causing the dissipation of the proton-driven potential (27). Although different antimicrobial peptides achieve this ultimate end, their target mechanisms are different. Currently, the detergent damage model and pore channel model are the two models that illustrate this mechanism (28). Both models are related to membrane damage and proton dynamic potential dissipation. Studies have shown that some antimicrobial peptides cause damage to bacterial cell membranes, decrease the fluidity of the membrane, and/or cause loss of its semi-permeability. Ultimately, resulting in leakage of intracellular electrolytes and nucleic acids to the outside of the cell, simultaneously affecting the various metabolic pathways of the cell. Interference with the regulation of osmotic pressure inside and outside the cell eventually leads to cell death (29). Potassium and magnesium ions are two types of metal ions found in bacterial cells. Under normal circumstances, they exist stably inside the cells. When the cell membrane of the bacteria is either permeabilized or ruptured, a large amount of leakage will follow. Given this, most researchers use the detection of potassium ions or changes in magnesium ions concentration to assess cell membrane integrity (30).

### Effect of F1 on *E. coli* and *S. aureus* Phospholipids

**Figure 3:**
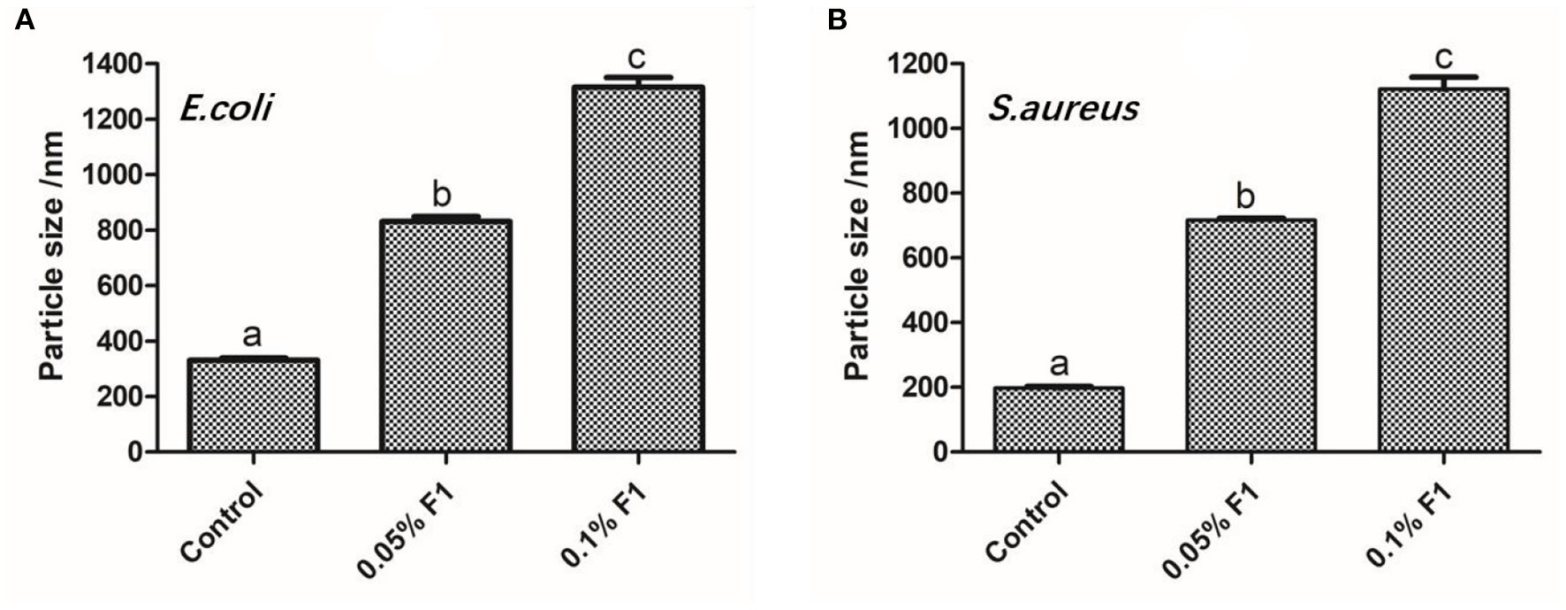
The effect of different concentrations of antibacterial peptide F1 on phospholipids of *E. coli* **(A)**, and *S. aureus* **(B)**. These letter represent statistically significant difference among the groups at *P* < 0.05.

To demonstrate that the antimicrobial peptide F1 directly reacts with bacterial liposomes, thus liposomes were extracted from both from *E. coli* and *S. aureus*. The liposomes made from the extracted lipids of *E. coli* and *S. aureus* have a degree of stability, and the particle size remained unchanged (~200 nm) after being left untouched for a period of time (Figure 3). After applying the antimicrobial peptide F1, the size of the liposomes significantly increased. After the antimicrobial peptide F1 application, the size of liposomes in the 0.1% antimicrobial peptide F1 group was significantly larger than that of the 0.05% antimicrobial peptide F1 liposomes. This difference indicated that high concentrations of antimicrobial peptide F1 interfered with liposomal stability. This instability was caused by interacting with liposomes to increase their size. The increase in particle size of *S. aureus* liposomes was larger than that of *E. coli* liposomes, indicating that antimicrobial peptide F1 had different degrees of damage to liposomes of different strains, indicating that F1 has a certain selectivity for liposome damage.

### Effect of Antibacterial Peptide F1 on Liposomes

Calcein has self-quenching properties. When high concentrations of calcein are found inside liposomes, the fluorescence value is low. When antibacterial peptides damage liposomes, calcein leakage starts and resulting in an increased fluorescence. The internal strength increases with the degree of leakage; the molecular diameter is smaller than that of probes such as FITC, which is suitable for liposomes with small particle size (31).

**Figure 4:**
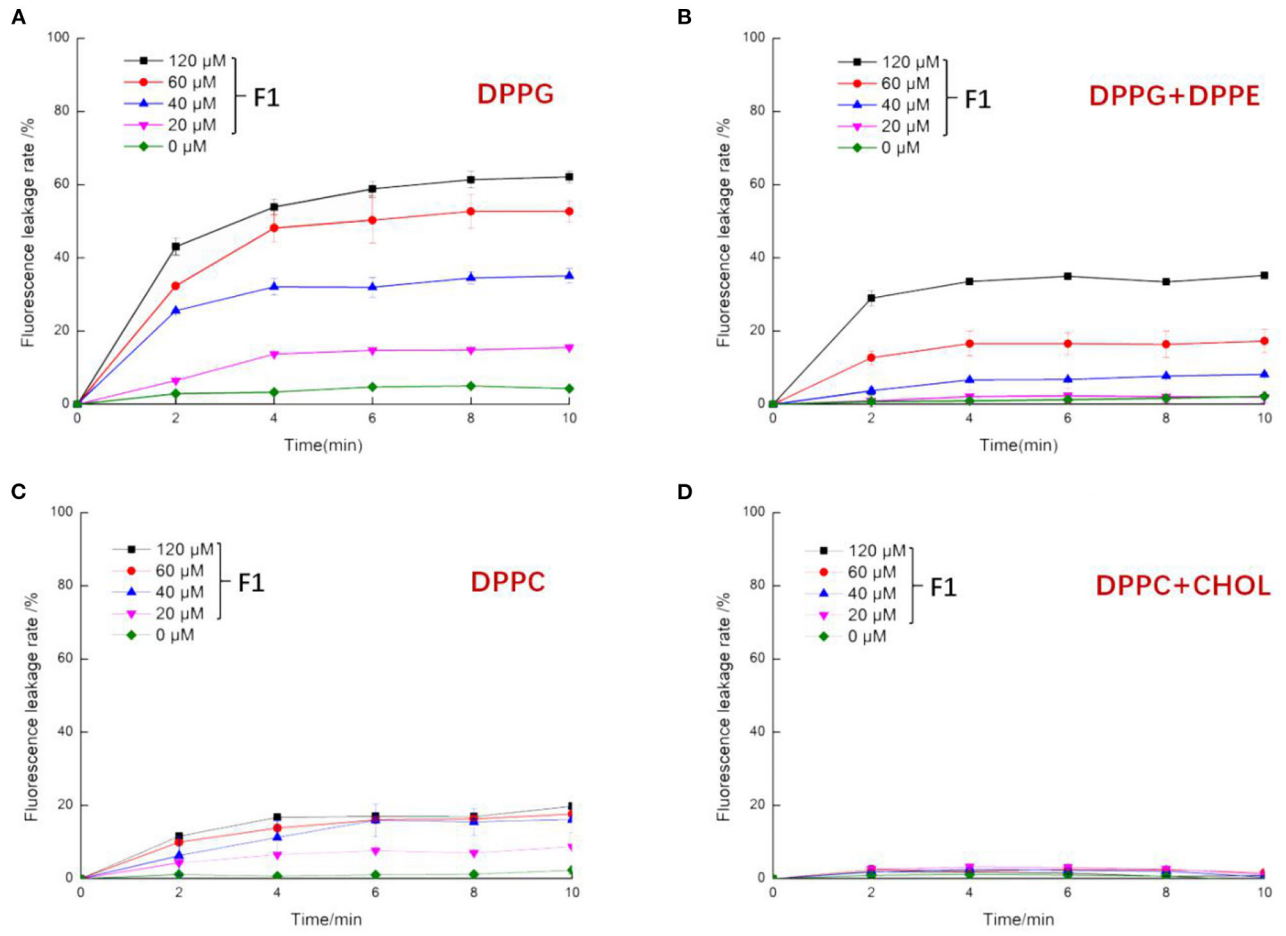
The effect of antibacterial peptide F1 on artificial liposomes. Composition of artificial liposomes was as follows: **(A)** DPPG (100%), **(B)** DPPG (30%) + DPPE (70%), **(C)** DPPC (100%), and **(D)** DPPC (90%) +CHOL (10%).

According to the fluorescence working curve of calcein shown in Supplementary Figure 1, it is clear that the fluorescence value of calcein has a concentration range that showed a linear change. Taking the level of fluorescence leakage after adding 10% Triton X-100 as an indicator of the complete leakage of liposomes. More specifically, it has a good linear relationship across a fluorescence intensity range of 0–400,000. This quantitatively reflects the leakage level of the probe. When the concentration exceeds 3 μg/mL, the fluorescence intensity exceeds this range and the fluorescence value no longer significantly increases. As shown in Figures 4A,B, after treatment with F1, the above two liposomes had strong fluorescence leakage. Moreover, the leakage degree increased with increasing F1 concentration. The leakage rate of DPPG liposomes under the same F1 concentration was significantly greater than that of the DPPG + DPPE liposomes. When the liposomes contained DPPE molecules, the efficiency of F1 was reduced. This may be because the addition of DPPE molecules (without charge) reduces the overall negative charge. As shown in the aforementioned figures, the fluorescence leakage after F1 acted on the liposomes was very fast. More specifically, a leakage level of more than 80% was observed within the time span of 2 min; over the following 2–10 min, subsequent leakage rate increase was not very high. This indicated that the interaction between the antimicrobial peptide F1 and the phospholipid membrane occurred quickly and when F1 contacted other liposomes, the amount of leakage increased. DPPC and cholesterol are the main components of mammalian cell membranes (32). DPPC molecules are not charged, and cholesterol has a protective effect on the stability of phospholipid membranes. As shown in Figures 4C,D, the effect of F1 on DPPC liposomes was significantly smaller than that on DPPG and DPPG + DPPE. The effect on DPPC + CHOL was smaller, and the fluorescence leakage rate in these liposomes never exceeded 10%. This reduction in permeability was due to the cholesterol molecules introducing conformational ordering of the lipid chains and creating a denser and more rigid barrier for the calcein to cross (33).

### TEM Observation of the Effect of F1 on the Phospholipid Membrane

**Figure 5:**
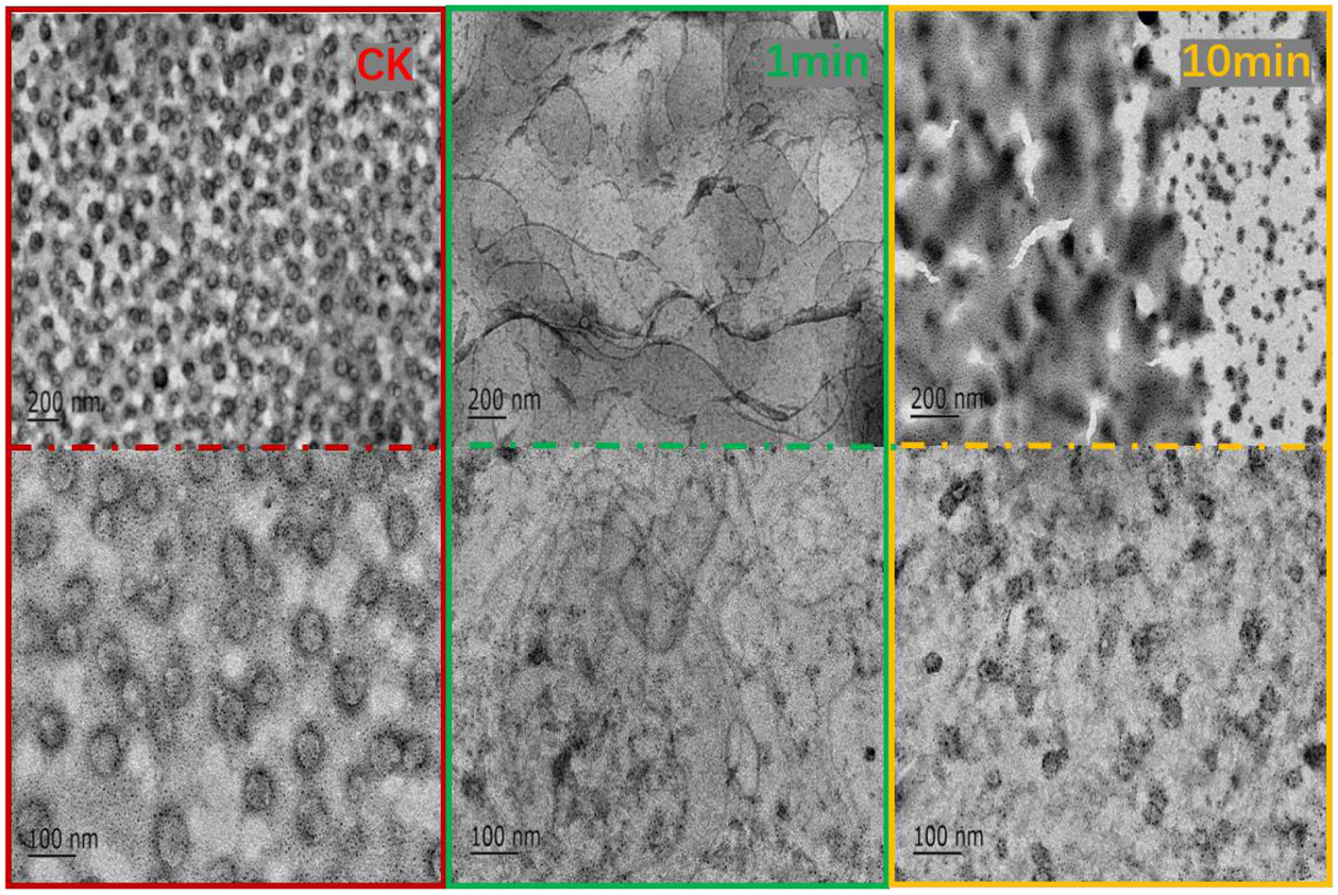
TEM observation of the effect of antibacterial peptide F1 on phospholipid membrane at 1 and 10 min.

TEM is commonly used to characterize the internal structure of nanoemulsions (34). Nano-sized liposomes are dispersed in the buffer and their integrity is observable using transmission electron microscope (TEM). As shown in Figure 5, before adding F1, liposomes were found in a regular and uniform spherical shape as liposomes primarily appeared as unilamellar spherical vesicles consisting of a lipid bilayer (higher density bands) surrounding an aqueous core (same density as exterior aqueous phase) (35). One min after adding the antimicrobial peptide F1 to the system, the liposomes were fused and presented as a large liquid phospholipid membrane. At this point, the phospholipid bilayer completely lost its structure. After the antimicrobial peptide F1 interacted with the phospholipid membrane for 10 min, the phospholipid membrane broke into an irregular dispersion. When examined closely, it was clear that the original liposome became a transparent phospholipid membrane structure. Although the liposomes were clearly fused, they maintained a membranous structure. When acting upon for a length of time, the phospholipid membrane ruptured, and the precipitate accumulated. This led to increased agglomeration between liposomes over time, the liposome membrane ruptured and the average particle size increased.

Conclusively, the study's results showed that the antimicrobial peptide F1 disturbed the structure of the phospholipid membrane, thereby destroying the cell membrane structure of the tested bacteria and causing the cell body contents to leak out ultimately resulting in cell death.

### Effect of F1 on Spreading Phospholipid Membrane

**Figure 6:**
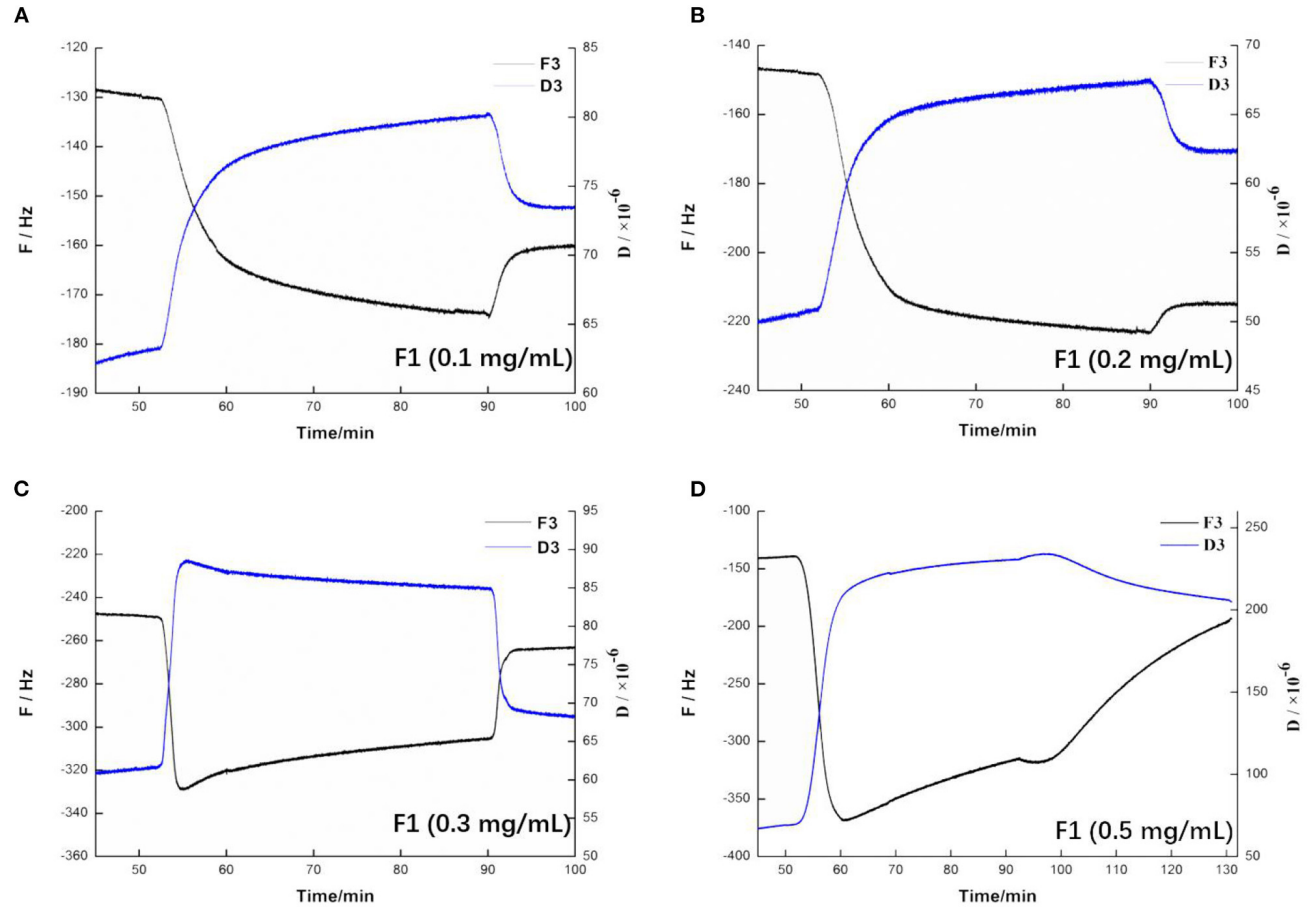
Changing trend of frequency and dissipation at three times the frequency on the membrane after applying antimicrobial peptide F1. Different concentrations of antimicrobial peptide F1: **(A)** 0.1 mg/mL, **(B)** 0.2 mg/mL, **(C)** 0.3 mg/mL, and **(D)** 0.5 mg/mL.

QCM-D is an emerging technology based on quartz crystal microbalance (QCM) technology (36). QCM-D monitors the frequency (F) and energy dissipation (D) of the quartz crystal surface in real time, thereby monitoring the dynamics of the surface of the membrane solid support membrane and measuring the quality and viscoelasticity of the phospholipid membrane (37). As shown in Figure 6A, before adding F1, the frequency and dissipation of the wafer surface were relatively stable, and the phospholipid membrane had reached a fairly stable state. Fifty minutes min after F1 had been added, the frequency and dissipation fluctuated greatly. The frequency significantly decreased, indicating that the quality of the membrane surface increased. Moreover, the membrane surface quality continued to increase slowly after stopping the flow of F1. After allowing the flow into the buffer to rest, the frequency and the dissipation value both recovered and quickly stabilized. The final frequency and dissipation values had a certain difference from before flowing into F1, indicating that both the quality of the membrane surface and its viscoelasticity had increased. Taken together, these results showed that this concentration of F1 was adsorbed on the surface of the phospholipid membrane, which changed the physical parameters of the membrane surface. As shown in Figure 6B, the change on the surface of the phospholipid membrane at 0.2 mg/mL F1 was almost the same as that observed at 0.1 mg/mL. However, the frequency of the membrane surface was more notably changed. This indicated that F1 adsorbed more on the membrane surface, but did not show any obvious destructive effects. As shown in Figure 6C, when 0.3 mg/mL F1 was added, the frequency of the membrane surface also decreased, and the dissipation phenomenon increased. When F1 was continuously in contact with the phospholipid membrane, the frequency of the membrane surface began to slowly rise, and dissipation was slow and had decreased. At this stage, this indicated that the membrane mass was reduced, the viscoelasticity was reduced, and/or the membrane was thinned. After flowing into the buffer, the frequency increased and was closer to the initial value than when the concentration was low. This indicated that more material was washed away on the surface of the wafer. It may be that the phospholipid membrane was damaged due to the action of F1, causing part of the phospholipid molecules to fall off. As shown in Figure 6D, after adding 0.5 mg/mL of F1, both the mass and viscoelasticity of the membrane increased rapidly during the adsorption period. During the standing process, the frequency significantly decreased, which indicated that the mass of F1 decreased after interacting with the phospholipid membrane. Moreover, the D value also slowly increased (4).

**Figure 7:**
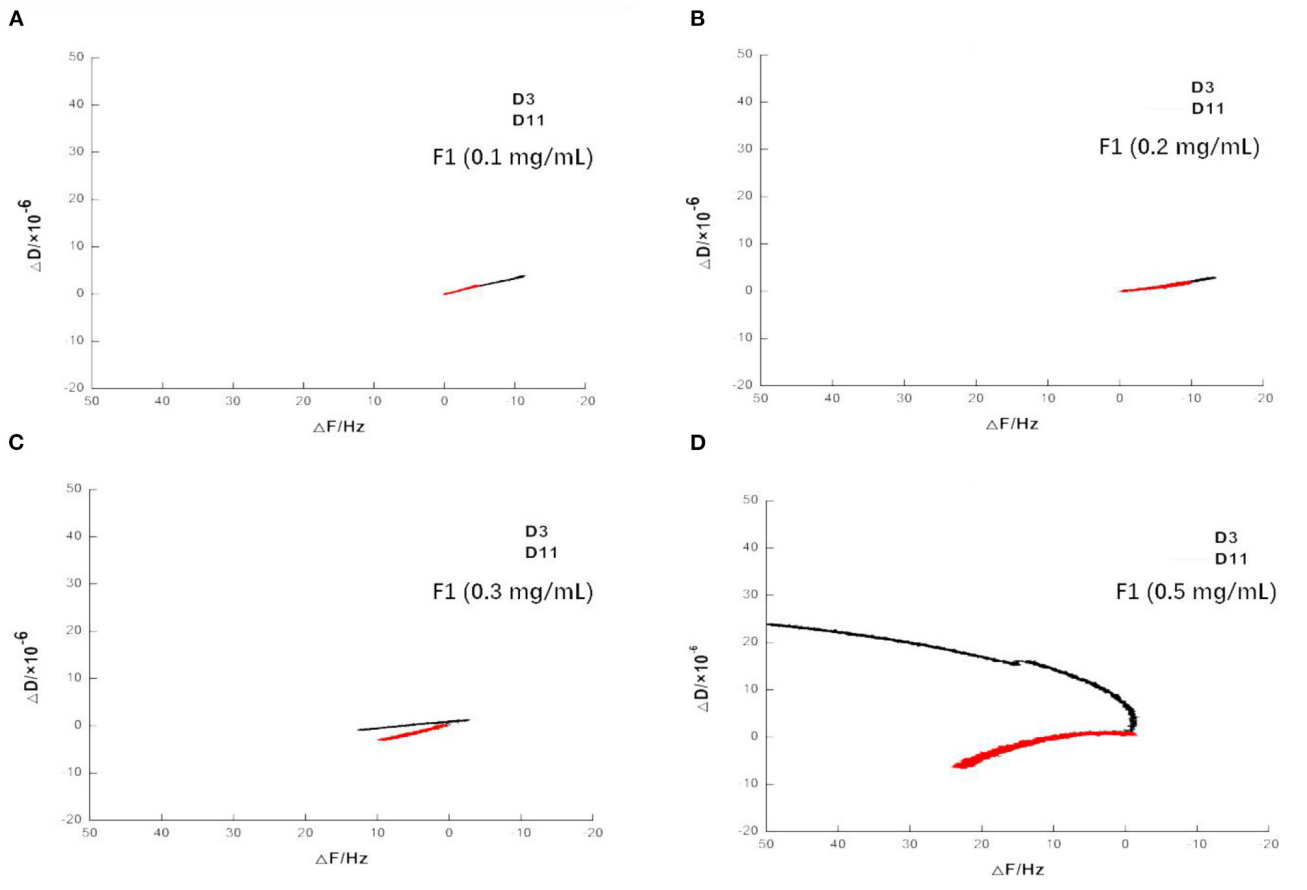
Variation of dissipation on spreading phospholipid membrane at frequency of 3 and 11 times after applying antimicrobial peptide F1. Different concentrations of antimicrobial peptide F1: **(A)** 0.1 mg/mL, **(B)** 0.2 mg/mL, **(C)** 0.3 mg/mL, and **(D)** 0.5 mg/mL.

**Figure 8:**
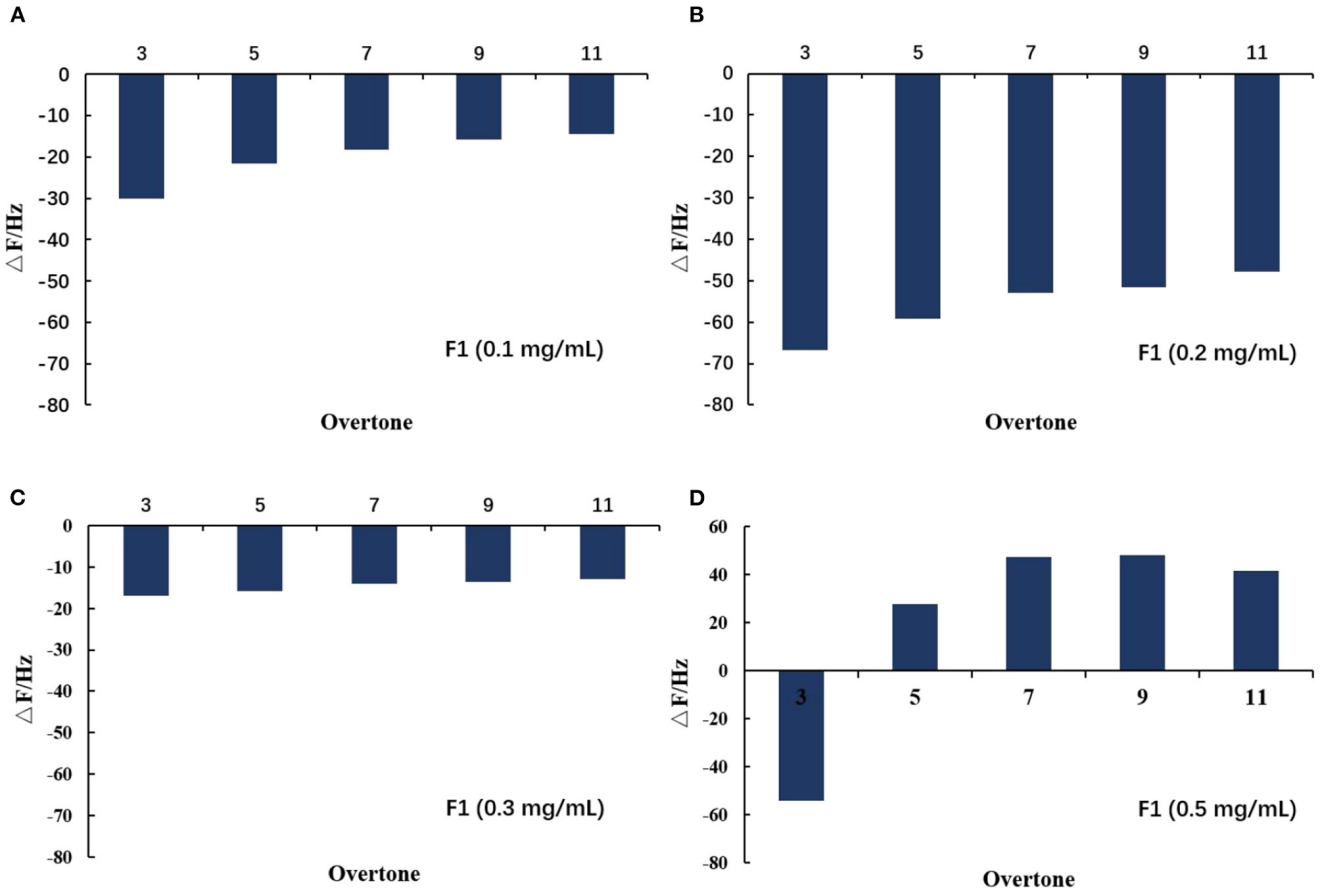
Variation of film value on spreading phospholipid membrane at different octaves after applying antimicrobial peptide F1. Different concentrations of antimicrobial peptide F1: **(A)** 0.1 mg/mL, **(B)** 0.2 mg/mL, **(C)** 0.3 mg/mL, and **(D)** 0.5 mg/mL.

After adding the buffer, the frequency increased to a large degree, indicating a large amount of damage to the phospholipid membrane and the washing away of a large amount of material on the wafer. As shown in Figures 7A–D, when 0.1 and 0.2 mg/mL of F1 were individually applied, the dissipation at 3 octave and 11 octave increased with increasing frequency. Comparatively, at 0.3 mg/mL, the F and D values began to change. When the concentration reached 0.5 mg/mL, its change law became more complicated and the curve of the 3 octave frequency changed to the upper left direction. The slope of the curve was first larger and then smaller, and the curve of the 11 octave frequency changed to the lower left. Taken together, these results showed that the viscoelasticity of the phospholipid membrane on the wafer surface varied at different depths (14). As shown more intuitively in Figures 8A–D, the value of the membrane changed at different frequency doublings. At lower F1 concentrations, the surface quality of the film increased; notably, this increase became larger with increasing concentration. At the same concentration, the amount of mass increase decreased with an increase of the frequency doubling. As shown, the change on the surface of the film was greater, while the internal impact was less. With increasing F1 concentration, the mass change was not proportional to the concentration, but began to decrease. After applying 0.3 mg/mL F1, the frequency of each frequency doubling was reduced; that is, the mass increased, but the increase was <20 Hz. This was notably <0.2 mg/mL. When the concentration reached 0.5 mg/mL, the frequency still decreased at the three times the frequency, while the frequency of five to eleven-times was significantly increased. Taken together, this indicated that the internal quality of the membrane was decreased and the membrane was no longer intact after F1 application.

In summary, the results of the QCM-D experiment showed that different F1 concentrations had different effects on the surface of the phospholipid membrane. When either 0.1 or 0.2 mg/mL F1 was applied over the membrane surface, adsorption occurred. Moreover, the amount of adsorption increased with increasing F1 concentration. However, when F1 with a concentration higher than 0.3 mg/mL was applied onto the surface of the phospholipid membrane, it caused significant damage to the phospholipid membrane. At 0.3 mg/mL of F1, the membrane surface quality was slightly reduced. At 0.5 mg/mL of F1, the membrane quality was significantly reduced, but the viscoelasticity increased. The changes in the properties of the membrane surface and the interior of the membrane were not fully synchronized, but as the concentration increased, the amount of molecular aggregation also increased and affected the membrane integrity.

## Conclusions

Based on the study's findings, antimicrobial peptide F1 exhibited potential to inhibit the growth of both Gram-negative and Gram-positive bacteria. Specifically, antimicrobial peptide F1 mainly interacts with the bacterial liposome membrane, causing the membrane to lose viscoelasticity and destroying its structure leading to destroy the structure of the entire cell membrane, subsequently causing the cell content's leakage and killing of the bacteria. This study showed that F1 exhibited promising antimicrobial activities and could possibly be used to enhance antimicrobial status of commercial edibles and also to develop organic antimicrobials to counter foodborne and of medical importance pathogens.

## Data Availability Statement

The raw data supporting the conclusions of this article will be made available by the authors, without undue reservation.

## Author Contributions

QW, BP, YC, and XZ designed the experiment. QW, BP, FC, and JM conducted the experiments. QW, BP, KF, and MS did the experimental analysis, collection, and analysis of the data. QW, BP, JL, and Abdullah wrote and revised the manuscript. All authors contributed to the article and approved the submitted version.

## Funding

This work was supported by the National Natural Science Foundation of China (No. 31972078), Program for Guangdong Introducing Innovative and Entrepreneurial Teams (2019ZT08N291) and Clinical Chinese Medicine Plateau Discipline Construction Project of Shanghai Pudong New Area Health Committee (PDZY-2018-0604).

## Conflict of Interest

BP was employed by company Guangdong Haitian Innovation Technology Co., Ltd. FC was employed by company Evonik Rexim Nanning Co. The remaining authors declare that the research was conducted in the absence of any commercial or financial relationships that could be construed as a potential conflict of interest.

## Publisher's Note

All claims expressed in this article are solely those of the authors and do not necessarily represent those of their affiliated organizations, or those of the publisher, the editors and the reviewers. Any product that may be evaluated in this article, or claim that may be made by its manufacturer, is not guaranteed or endorsed by the publisher.

## Supplementary Material

Supplementary Figure 1 Fluorescence working curve of calcein.

## References

[1] Mason AJ Marquette A Bechinger B. Zwitterionic phospholipids and sterols modulate antimicrobial peptide-induced membrane destabilization. Biophys J. (2007) 93:4289–99. 10.1529/biophysj.107.116681 17766347 PMC2098721

[2] Lombardi L Stellato MI Oliva R Falanga A Galdiero M Petraccone L. Antimicrobial peptides at work: interaction of myxinidin and its mutant WMR with lipid bilayers mimicking the *P. aeruginosa* and *E coli* membranes. Sci Rep. (2017) 7:44425. 10.1038/srep44425 28294185 PMC5353584

[3] Bellomio A Oliveira RG Maggio B Morero RD. Penetration and interactions of the antimicrobial peptide, microcin J25, into uncharged phospholipid monolayers. J Colloid Interface Sci. (2005) 285:118–24. 10.1016/j.jcis.2004.11.025 15797404

[4] Wang KF Nagarajan R Camesano TA. Differentiating antimicrobial peptides interacting with lipid bilayer: molecular signatures derived from quartz crystal microbalance with dissipation monitoring. Biophys Chem. (2015) 196:53. 10.1016/j.bpc.2014.09.003 25307196

[5] Li J Wang XL Zhang T Wang CL Huang ZJ Lou X. A review on phospholipids and their main applications in drug delivery systems. Asian J Pharm Sci. (2015) 10:81–98. 10.1016/j.ajps.2014.09.004

[6] Vanounou S Parola AH Fishov I. Phosphatidylethanolamine and phosphatidylglycerol are segregated into different domains in bacterial membrane. A study with pyrene-labelled phospholipids. Mol Microbiol. (2003) 49:1067–79. 10.1046/j.1365-2958.2003.03614.x 12890029

[7] Strahl H Bürmann F Hamoen LW. The actin homologue MreB organizes the bacterial cell membrane. Nat Commun. (2014) 5:3442. 10.1038/ncomms4442 24603761 PMC3955808

[8] Rathinakumar R Wimley WC. Biomolecular engineering by combinatorial design and high-throughput screening: small, soluble peptides that permeabilize membranes. J Am Chem Soc. (2008) 130:9849–58. 10.1021/ja8017863 18611015 PMC2582735

[9] Chen F Lee M Huang HW. Sigmoidal concentration dependence of antimicrobial peptide activities: a case study on alamethicin. Biophys J. (2002) 82:908–14. 10.1016/S0006-3495(02)75452-0 11806932 PMC1301899

[10] Russell AL Kennedy AM Spuches AM Venugopal D Bhonsle JB Hicks RP. Spectroscopic and thermodynamic evidence for antimicrobial peptide membrane selectivity. Chem Phys Lipids. (2010) 163:488–97. 10.1016/j.chemphyslip.2010.03.009 20362562

[11] Boschkova K Stålgren JJR. Cationic and nonionic surfactant adsorption on thiol surfaces with controlled wettability. Langmuir. (2002) 18:6802–6. 10.1021/la011774b

[12] Liu SX Kim J. Study of adsorption kinetics of surfactants onto polyethersulfone membrane surface using QCM-D. Desalination. (2009) 247:355–61. 10.1016/j.desal.2008.08.002

[13] Chen KL Bothun GD. Nanoparticles meet cell membranes: probing nonspecific interactions using model membranes. Environ Sci Technol. (2014) 48:873–80. 10.1021/es403864v 24341906

[14] Melendrez D Jowitt T Iliut M Verre AF Goodwin S Vijayaraghavan A. Adsorption and binding dynamics of graphene-supported phospholipid membranes using the QCM-D technique. Nanoscale. (2018) 10:2555–567. 10.1039/C7NR05639G 29349454

[15] Miao J Liu G Ke C Fan W Li C Chen Y. Inhibitory effects of a novel antimicrobial peptide from kefir against *Escherichia coli*. Food Control. (2016) 65:63–72. 10.1016/j.foodcont.2016.01.023

[16] Miao J Peng W Liu G Chen Y Chen F Cao Y. Biopreservative effect of the natural antimicrobial substance from Lactobacillus paracasei subsp. tolerans FX-6 on fresh pork during chilled storage. Food Control. (2015) 56:53–6. 10.1016/j.foodcont.2015.03.013

[17] Miao JY Guo HX Ou YW Liu G Fang X Liao ZL. Purification and characterization of bacteriocin F1, a novel bacteriocin produced by Lactobacillus paracasei subsp. tolerans FX-6 from Tibetan kefir, a traditional fermented milk from Tibet, China. Food Control. (2014) 42:48–53. 10.1016/j.foodcont.2014.01.041

[18] Duan H Jin S Zhang Y Li F Xiang J. Granulocytes of the red claw crayfish Cherax quadricarinatus can endocytose beads, *E. coli* and WSSV, but in different ways. Dev Compar Immunol. (2014) 46:186–93. 10.1016/j.dci.2014.04.006 24747430

[19] Dennison SR Morton LH Brandenburg K Harris F Phoenix DA. Investigations into the ability of an oblique alpha-helical template to provide the basis for design of an antimicrobial anionic amphiphilic peptide. FEBS J. (2006) 273:3792–803. 10.1111/j.1742-4658.2006.05387.x 16911526

[20] Lee W Lee DG. Fungicidal mechanisms of the antimicrobial peptide Bac8c. Biochimica et Biophysica Acta. (2015) 1848:673–9. 10.1016/j.bbamem.2014.11.024 25434926

[21] Hugonin L Vukojević V Bakalkin G Gräslund A. Membrane leakage induced by dynorphins. FEBS Lett. (2006) 580:3201–5. 10.1016/j.febslet.2006.04.078 16697372

[22] Cho NJ Frank CW Kasemo B Hook F. Quartz crystal microbalance with dissipation monitoring of supported lipid bilayers on various substrates. Nat Protoc. (2010) 5:1096–106. 10.1038/nprot.2010.65 20539285

[23] Zasadzinski JA. Transmission electron microscopy observations of sonication-induced changes in liposome structure. Biophys J. (1986) 49:1119–30. 10.1016/S0006-3495(86)83741-9 3719073 PMC1329696

[24] Miao J Zhou J Liu G Chen F Chen Y Gao X. Membrane disruption and DNA binding of Staphylococcus aureus cell induced by a novel antimicrobial peptide produced by *Lactobacillus paracasei* subsp. tolerans FX-6. Food Control. (2016) 59:609–13. 10.1016/j.foodcont.2015.06.044

[25] Slavin YN Asnis J Hafeli UO Bach H. Metal nanoparticles: understanding the mechanisms behind antibacterial activity. J Nanobiotechnol. (2017) 15:65. 10.1186/s12951-017-0308-z 28974225 PMC5627441

[26] Mai-Prochnow A Clauson M Hong J Murphy AB. Gram positive and Gram negative bacteria differ in their sensitivity to cold plasma. Sci Rep. (2016) 6:38610. 10.1038/srep38610 27934958 PMC5146927

[27] Bauer R Dicks LMT. Mode of action of lipid II-targeting lantibiotics. Int J Food Microbiol. (2005) 101:201–16. 10.1016/j.ijfoodmicro.2004.11.007 15862882

[28] Montville TJ Winkowski K Ludescher RD. Models and mechanisms for bacteriocin action and application. Int Dairy J. (1995) 5:797–814. 10.1016/0958-6946(95)00034-8

[29] Tang Y Shi Y Zhao W Hao G Le G. Discovery of a novel antimicrobial peptide using membrane binding-based approach. Food Control. (2009) 20:149–56. 10.1016/j.foodcont.2008.03.006

[30] Lee M Yang P Charron NE Hsieh M Chang Y Huang HW. Comparison of the effects of daptomycin on bacterial and model membranes. Biochemistry. (2018) 57:5629–39. 10.1021/acs.biochem.8b00818 30153001

[31] Shimanouchi T Ishii H Yoshimoto N Umakoshi H Kuboi R. Calcein permeation across phosphatidylcholine bilayer membrane: Effects of membrane fluidity, liposome size, and immobilization. Colloids Surf B Biointerfaces. (2009) 73:156–60. 10.1016/j.colsurfb.2009.05.014 19560324

[32] van der Veen JN Kennelly JP Wan S Vance JE Vance DE Jacobs RL. The critical role of phosphatidylcholine and phosphatidylethanolamine metabolism in health and disease. Biochimica et Biophysica Acta. (2017) 1859:1558–72. 10.1016/j.bbamem.2017.04.006 28411170

[33] Moorcroft SCT Roach L Jayne DG Ong ZY Evans SD. Nanoparticle-loaded hydrogel for the light-activated release and photothermal enhancement of antimicrobial peptides. ACS Appl Mater Interfaces. (2020) 12:24544–54. 10.1021/acsami.9b22587 32312040

[34] Hoffmann I Michel R Sharp M Holderer O Appavou M Polzer F. Softening of phospholipid membranes by the adhesion of silica nanoparticles – as seen by neutron spin-echo (NSE). Nanoscale. (2014) 6:6945–52. 10.1039/C4NR00774C 24838980

[35] Fox CB Mulligan SK Sung J Dowling QM Fung HW Vedvick TS. Cryogenic transmission electron microscopy of recombinant tuberculosis vaccine antigen with anionic liposomes reveals formation of flattened liposomes. Int J Nanomed. (2014) 9:1367–77. 10.2147/IJN.S56582 24648734 PMC3956628

[36] Tonda-Turo C Carmagnola I Ciardelli G. Quartz crystal microbalance with dissipation monitoring: a powerful method to predict the *in vivo* behavior of bioengineered surfaces. Front Bioeng Biotechnol. (2018) 6:158. 10.3389/fbioe.2018.00158 30425985 PMC6218436

[37] Wang KF Nagarajan R Camesano TA. Antimicrobial peptide alamethicin insertion into lipid bilayer: a QCM-D exploration. Colloids Surf B Biointerfaces. (2014) 116:472–81. 10.1016/j.colsurfb.2014.01.036 24561501

