# Supplementary figures and images for: Effects of Antibacterial Peptide F1 on Bacterial Liposome Membrane Integrity
Source: Front Nutr. 2021 Nov 12;8:768890. doi: 10.3389/fnut.2021.768890 (PMC8633404; doi:10.3389/fnut.2021.768890)

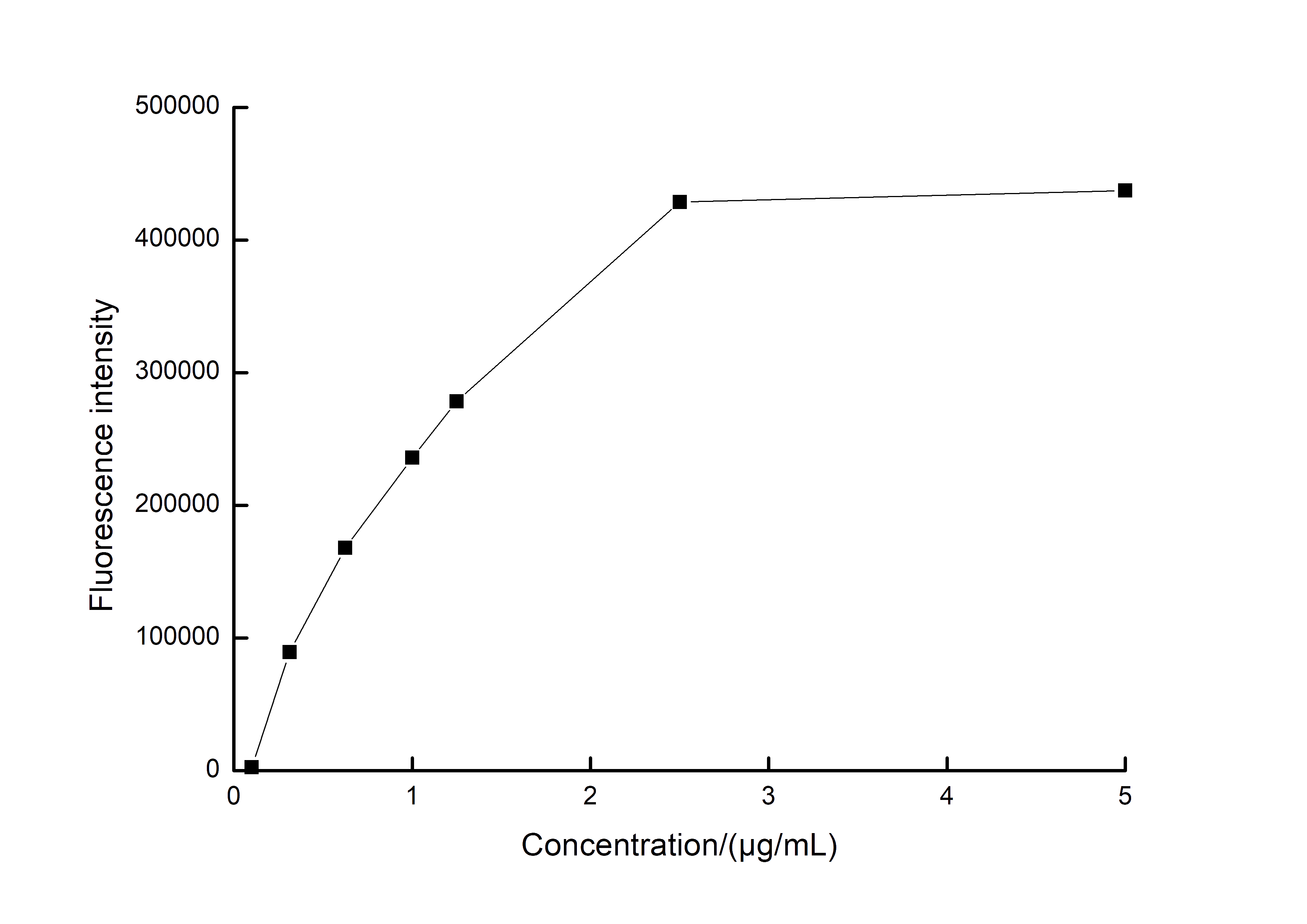

Supplement: Supplementary Figure 1 — Fluorescence working curve of calcein. [file Image_1.TIF]
